# Urinary Steroid Profile in Elite Female Athletes in Relation to Serum Androgens and in Comparison With Untrained Controls

**DOI:** 10.3389/fphys.2021.702305

**Published:** 2021-08-30

**Authors:** Emma Eklund, Alexander Andersson, Lena Ekström, Angelica Lindén Hirschberg

**Affiliations:** ^1^Department of Women’s and Children’s Health, Division of Neonatology, Obstetrics and Gynecology, Karolinska Institutet, Stockholm, Sweden; ^2^Department of Laboratory Medicine, Division of Clinical Pharmacology, Karolinska Institutet, Karolinska University Hospital, Stockholm, Sweden; ^3^Department of Gynecology and Reproductive Medicine, Karolinska University Hospital, Stockholm, Sweden

**Keywords:** serum androgens, urinary steroid profile, athlete biological passport, exercise, female athlete

## Abstract

**Introduction:**

In female athletes, the interpretation of doping tests is complex due to hormonal variations during the menstrual cycle and hormonal contraceptive use, both influencing the urinary steroid profile. Exercise is suggested to affect circulating steroid hormone levels, and in women, the urinary steroid profile differs between in competition testing and out of competition testing. No previous study has investigated the relationship between amount of exercise and the urinary steroid profile in female elite athletes.

**Purpose:**

To compare the urinary steroid profile between female Olympic athletes and age- and BMI-matched untrained controls, and to study the urinary steroid profile in relation to serum hormones and amount of exercise.

**Methods:**

In this cross-sectional study conducted at the Women’s Health Research Unit, Karolinska University Hospital, Stockholm, 94 female elite athletes and 86 untrained controls were included. Serum estrogens and testosterone and the urinary steroid profile were analyzed by liquid chromatography–tandem mass spectrometry and gas chromatography-tandem mass spectrometry, respectively. Exercise hours/week were evaluated by questionnaire.

**Results:**

Although serum steroid hormones were comparable between groups, the athletes demonstrated approximately 30% lower urinary steroid metabolites of testosterone, epitestosterone, androsterone, etiocholanolone, 5α-androstan-3α, 17β-diol, and 5β-androstan-3α, 17β-diol compared to the controls. The urinary steroid metabolites correlated positively with serum steroid hormones. In the athletes, urinary steroid metabolites: androsterone (*r*_*s*_ = −0.28, *p* = 0.007), epitestosterone (*r*_*s*_ = −0.22, *p* = 0.034), 5αAdiol (*r*_*s*_ = −0.31, *p* = 0.002) and testosterone (*r*_*s*_ = −0.24, *p* = 0.026), were negatively correlated with amount of training (hours per week).

**Conclusion:**

The urinary concentrations of steroid metabolites were lower in elite athletes than in sedentary controls, although serum steroids were comparable between groups. Moreover, exercise time was negatively associated with the urinary concentrations. Our findings suggest alternative excretion routes of androgens in the athletes related to training.

## Introduction

Since 2014, the athlete biological passport (ABP) has been used to identify doping with endogenous anabolic steroids, such as testosterone (T). The urinary concentrations of T, its isomer epitestosterone (E), and the T metabolites, androsterone (A), etiocholanolone (Etio), 5α-androstanediol (5αAdiol), and 5β-androstanediol (5βAdiol) are analyzed by gas chromatography-tandem mass spectrometry (GC-MS/MS). These concentrations are combined into five ABP ratios (T/E, A/Etio, 5αAdiol/E, 5αAdiol/5βAdiol, and A/T), and an adaptive Bayesian algorithm calculates individual reference thresholds (Sottas et al., 2010; Sottas and Vernec, 2012). An atypical passport finding is obtained when a sample in the passport goes outside the individually calculated reference ranges, which may trigger a confirmatory isotope ratio mass spectrometry analysis to identify if testosterone is of endogenous or exogenous origin (Wada Wada Technical Document, 2021a, b). The urinary profile is analyzed after the urine has been hydrolyzed with β-glucuronidase and consequently, it is the unconjugated, as well as the glucuronidated fractions that are quantified. Even though glucuronidation is the main excretion route of androgens, the ABP metabolites are also to a lesser extent excreted as sulfate-conjugates (Rane and Ekstrom, 2012; Schiffer et al., 2019).

We, and others, have previously shown that implementation of the ABP increases the chance to detect testosterone intake in men as compared to traditional population based cut-off values after administration of low dose of testosterone (Strahm et al., 2015; Mullen J. et al., 2017; Nair et al., 2020). Furthermore, in women the longitudinal ABP approach is superior to the population-based thresholds to detect administered T, however, not all women are identified as having atypical findings (Handelsman and Bermon, 2019; Knutsson et al., 2020; Salamin et al., 2020). It has been shown that after 10 weeks of transdermal T application in healthy women, only 40% were identified as having atypical passport findings (Knutsson et al., 2020) even though their serum T levels were elevated to concentrations associated with performance enhancing effects (Hirschberg et al., 2020). As a supplementary method, the serum concentration of T, androstenedione and dihydrotestosterone may be co-monitored (Salamin et al., 2020) and subsequently there is an interest to understand the relation between the serum steroid concentrations and urinary excretion rate of the ABP metabolites.

It is well known that hormones fluctuate during the menstrual cycle. For example, urinary E is at the highest concentrations in the luteal phase, whereas the other ABP metabolites show minor fluctuations during a menstrual cycle. These variations result in larger individual ABP-thresholds in women (Schulze et al., 2020). Other challenges associated with ABP interpretation in female athletes include the use of hormonal contraceptives (HC) (Schulze et al., 2014; Ekström et al., 2019), and the impact of genetic polymorphisms in UDP-glucuronosyltransferases (UGTs), such as UGT2B17 (Schulze et al., 2014). The time of sampling may also be pivotal for test-results. In a large compilation of ABP data from over 11,000 Swedish and Norwegian athletes, both the intra- and inter-individual variations for all ABP ratios were larger in women than men. Furthermore, women demonstrated 65% higher T excretion in competition (IC) as compared to out of competition (OOC), whereas men’s urinary steroid profile did not differ to the same extent between IC and OOC testing (Mullen et al., 2020). In women, a great part of the androgen production occurs in the adrenal gland stimulated by adrenocorticotropic hormone (ACTH) from the pituitary gland (Burger, 2002; Schiffer et al., 2019). Since physiological stress stimulates ACTH secretion and in turn androgen and cortisol from the adrenal gland (Schiffer et al., 2019) this may explain the higher T excretion IC found in women.

In addition, previous studies suggest that exercise affects the concentrations of circulatory hormones (Nindl et al., 2001; Enea et al., 2011), whereas there are no studies investigating the relationship between the amount of exercise and excretion rate of androgens. Therefore, we aimed to investigate the excretion profile of glucuronides and sulfate androgen conjugates in elite female athletes in relation to matched sedentary controls, and further to study the ABP urinary steroid profiles in relation to serum hormones, genetic variation in androgen metabolism and amount of exercise.

## Materials and Methods

### Study Population

The athletes participating in this study were part of a cohort of 106 Swedish female Olympic athletes, members of an Olympic team or part of the high-performance programs of the Swedish Olympic Committee (SOC). In addition, 117 healthy female controls [body mass index (BMI)- and age-matched, allowed a maximum of 2 h training/week and no prior participation in elite level sports] were recruited. A more detailed description of the study cohort is previously published (Eklund et al., 2017).

The present study population included 94 female Olympic athletes and 86 controls, from which urinary samples were available for analysis. Serum androgen levels and urinary sulphate levels were previously analyzed (Eklund et al., 2017, 2020) and are here presented for the same 94 athletes and 86 controls included in the current study.

The participants were investigated in connection with training camps or at the Women’s Health Research Unit, Karolinska University Hospital. Health status, training hours/week and gynecological data (hormonal contraceptive use, menstrual cycle data, pregnancies) were collected by a questionnaire. A blood sample and urinary samples were collected in a fasted, rested state between 07.00 and 10.00 h and stored at −20°C until further analyses. Blood and urine samples were collected randomly during the menstrual cycle.

The study was conducted according to the Declaration of Helsinki and was approved by the Regional Ethics Committee, Stockholm (EPN 2011/1426-32) and informed consent was acquired from all participants.

### Serum Hormonal Analyses

Serum T, estrone (E1), and estradiol (E2) were determined by liquid chromatography tandem mass spectrometry (LC-MS/MS) at the Endoceutics laboratory, Quebec, Canada, as previously described (Ke et al., 2014, 2015). Free androgen index (FAI) was calculated, testosterone nmol/L divided by sex hormone- binding globulin (SHBG) nmol/L × 100. Follicle stimulating hormone (FSH), luteinizing hormone (LH), SHBG and cortisol were analyzed by electrochemiluminescence immunoassay (ECLIA) using commercial kits from Roche Diagnostics AG (CH 6343 Rotkreuz, Switzerland) (Cobas8000), at the Department of Clinical Chemistry, Karolinska University hospital, Stockholm (Eklund et al., 2017). Detection limits and within and between assay coefficients of variation were for FSH 0.1 IU/L, 2.6 and 3.6%, for LH 0.1 IU/L, 1.2 and 2.0%, for SHBG 0.04 μg/mL, 1.3 and 2.1%, and for cortisol 1.5 nmol/L, 1.7 and 2.2%, respectively.

### Urinary Steroid Profile

The urinary levels (glucuronide and unconjugated fractions) of T, E, A, Etio, 5αAdiol, and 5βAdiol were determined with GC-MS/MS at the World Anti-doping agency (WADA) accredited anti-doping Laboratory at the Karolinska University Hospital, Huddinge, Stockholm as previously described (Mullen J.E. et al., 2017). Briefly, internal standard, phosphate buffer (pH 6.5) and β-glucuronidase from *E. coli* was added to 2 mL sample. The mix was incubated for 60 min at 50°C. Once cooled, the sample was extracted using potassium carbonate and methyl tert-butyl ether which was dried using sodium sulfate. After centrifugation the water phase was frozen, and the ether decanted into a fresh tube. The ether was evaporated under a gentle stream of nitrogen and placed in a desiccator. 100 μL derivatization reagent was added and left to react (incubated for 30 min at 50°C) to form tri-methyl silyl derivates. The sample was transferred to injection vials and injected to an Agilent 7890B gas chromatograph and 7000C Triple Quadrupole mass spectrometer.

Urinary sulphate metabolite levels [testosterone–sulphate (T–S), epitestosterone-sulphate (EpiT-S), androsterone-sulphate (ADT-S), and etiocholanolone-sulphate (Etio-S)], were analyzed using LC-MS/MS at the WADA accredited anti-doping Laboratory at the Karolinska University Hospital, Huddinge, Stockholm as previously described (Mullen J.E. et al., 2017; Eklund et al., 2020).

Specific gravity (SG) was measured with a Digital Refractometer to adjust for the dilution of the urine, using formula; *C*_corrected_ = *C*_measured_ × (1.020 − 1/SG − 1).

### Genotyping

Genomic DNA was extracted from whole blood using QIAmp DNA Blood Mini Kit (Qiagen). Twenty ng was used as template in 15 μL reactions using UGT2B17 copy number assay (#Hs03185327_cn, Life Technologies, Holland), and 2 × TaqMan Universal Master Mix II, no UNG (#4440043, Life Technology, Holland). The ubiquitously expressed RNase P (#4403326, Control Reagents, Life Technologies, Holland) was used as an endogenous reference gene for reaction quality control. Samples with RNase P signals but no UGT2B17 amplification were identified as del/del.

### Statistical Analyses

Continuous data was presented as mean ± SD when symmetrically distributed or as median and interquartile range (25th–75th percentile) otherwise. For comparisons between athletes and controls regarding HC use and the number of individuals with UGT2B17 del/del, the Pearson Chi-square test was applied. Mean serum hormones were compared between the groups using the student’s *t*-test when approximate normal distributions could be assumed. The Mann-Whitney *U*-test was used otherwise. Urinary androgen metabolites were not normally distributed and was therefore square root-transformed (testosterone, T/E ratio, and A/Etio) or log- transformed (all remaining urinary steroid metabolites and ratios) prior to parametric statistical tests. Concerning age, BMI, training hours per week and urinary androgen metabolites, comparison between groups were performed using the student’s *t*-test. Two-way ANOVA was applied to evaluate the potential impact of HC use when comparing urinary androgen metabolites and cortisol between athletes and controls (the interaction term group × HC use). In case of a significant interaction, differences between groups were tested with/and without HC use. Spearman correlation was used to evaluate association between variables. UGT2B17 del/del individuals were excluded in the Spearman correlations analyses between U-Testosterone and all serum androgens. *P*-values < 0.05 were considered statistically significant. Statistical analyses were performed using Statistica version 13 [TIBCO Software Inc. (2018)].

## Results

Age and BMI were comparable between groups. When comparing the frequency of HC use and UGT2B17 del/del individuals no significant differences were found between athletes and controls. As expected, the athletes had significantly higher amount of training per week (h/w) than the controls. As previously published, the athletes had significantly lower E1 levels compared to controls but no significant difference was found for serum T levels or FAI between athletes and controls (Eklund et al., 2017). The athletes demonstrated significantly lower urinary steroid metabolites (glucuronide and sulphate metabolites) compared to controls (Table 1).

**TABLE 1:** General characteristics, serum hormones and urinary androgen metabolites in female Olympic athletes and controls.

Parameter	Controls	Athletes
*n*	86	94
Age	26.3 ± 6.0	25.8 ± 5.5
BMI	22.0 ± 2.8	22.0 ± 1.9
HC use, *n* (%)	31 (36%)	36 (38%)
UGT2B17 del/del, *n* (%)	8 (9.4)	9 (9.7)
Amount of training (h/w)	0.7 ± 0.8	18.2 ± 5.8***
**Serum hormones**		
*n*	86	94
E1 (pg/mL)	47.4 (26.5–78.7)	34.9 (21.8–58.8)*
E2 (pg/mL)	55.5 (25.8–122.8)	35.6 (14.3–87.5)
T (pg/mL)	290.0 ± 105.6	284.9 ± 116.4
T (nmol/L)	1.0 ± 0.37	0.99 ± 0.40
FSH (IU/L)	4.0 (2.4–5.7)	4.6 (2.7–6.2)
LH (IU/L)	5.8 (3.4–7.9)	5.6 (2.4–8.9)
SHBG (nmol/L)	80.5 (62.0–129.0)	82.0 (57.0–117.0)
FAI	1.1 (0.6–1.8)	1.1 (0.6–1.9)
Cortisol (nmol/L)∧	516.0 ± 263.6	579.0 ± 216.3
**U-androgen metabolites**		
*n*	86	94
U-Testosterone-G (ng/mL)	6.90 (4.27–14.30)	4.59 (2.25–8.00)***
U-Testosterone-S (ng/mL)	1.77 (1.28–3.16)	1.55 (0.94–2.56)*
U-Epitestosterone-G (ng/mL)	10.99 (6.34–19.64)	6.09 (3.60–11.41)***
U-Epitestosterone-S (ng/mL)	5.79 (3.24–9.64)	2.69 (1.69–5.83)***
U-Androsterone-G (ng/mL)	3,386 (2,390–5,627)	2,178 (1,278–3,554)***
U-Androsterone-S (ng/mL)	756 (292–1,361)	519 (263–975)*
U-Etiocholanolone-G (ng/mL)	3,647 (2,504–4,838)	2,762 (1,769–4,139)**
U-Etiocholanolone-S (ng/mL)	273 (163–461)	255 (116–479)
U-5αAdiol-G (ng/mL)	33.4 (21.3–52.7)	19.6 (12.4–30.0)***
U-5βAdiol-G (ng/mL)	86.8 (53.0–197.1)	84.5 (39.1–132.8)*
T/E ratio∧	0.7 (0.5–1.1)	0.7 (0.4–1.3)
A/Etio ratio	1.0 (0.8–1.3)	0.8 (0.6–1.1)*
A/T ratio	458 (294–767)	428 (329–696)
5αAdiol/E	3.2 (1.9–5.0)	3.6 (2.4–4.8)
5αAdiol/5βAdiol	0.3 (0.2–0.6)	0.3 (0.2–0.5)

*Values presented as mean ± SD or median and interquartile range (25th–75th percentile).*

*A, androsterone; 5αAdiol, U-5α-Androstane-3α, 17β-diol; 5βAdiol, U-5β-Androstane-3α, 17β-diol; BMI, body mass index; E, epitestosterone; E1, estrone; E2, estradiol; Etio, etiocholanolone; FAI, free androgen index; FSH, follicular-stimulating hormone; h, hours; HC, hormonal contraceptive; LH, luteinizing hormone; SHBG, sex hormone-binding globulin; T, testosterone; S, sulphate metabolite; w, week.*

∧Two-way ANOVA indicated that HC use interacted with the comparison between groups.

*p < 0.05; **p < 0.01; ***p < 0.001.

In the athletes and controls not using HC, similar results were found when comparing urinary steroid metabolites (Supplementary Table 1). For the T/E ratio, two–way ANOVA indicated an interaction for HC use. Subgroup analyses found a significantly higher T/E ratio in controls using HC compared to controls not using HC [1.0 (0.7–1.5) vs. 0.6 (0.4–0.9), *p* < 0.001].

For cortisol, two- way ANOVA indicated that the differences between groups may be dependent on HC use. Comparison between groups demonstrated that in the subgroup of participants not using HC, athletes demonstrated significantly higher cortisol than controls (474.1 ± 131.2 vs. 376.4 ± 108.0, *p* = 0.004). In the subgroup using HC, no significant difference was found. As expected, HC users had significantly higher cortisol levels than non-users (athletes: 748.0 ± 220.7 vs. 474.1 ± 131.2, *p* = <0.0001, and controls: 759.1 ± 279.0 vs. 376.4 ± 108.0, *p* = <0.0001).

### Correlations Between Serum Hormones and Urinary Steroid Metabolites

Correlation analysis between the urinary androgen metabolites and serum androgens can be found in Table 2. In the subgroup of athletes and controls not using HC, similar correlations were found between serum hormones and urinary steroid metabolites (data not shown).

**TABLE 2:** Correlation matrix between serum androgens and urinary androgen metabolites in female Olympic athletes (*n* = 94) and controls (*n* = 86).

U-androgen metabolites	Serum androgens					
	E1	E2	T	FAI	FSH	LH
U-testosterone^#^						
Athletes	0.27*	ns	0.48***	0.72***	0.31**	0.26*
Controls	0.25*	0.31**	0.32**	0.48***	ns	0.37**
**Epitestosterone**						
Athletes	0.73***	0.65***	0.57***	0.61***	0.24*	0.54***
Controls	0.63***	0.65***	0.53***	0.65***	ns	0.55***
**U-Androsterone**						
Athletes	0.32**	0.22*	0.44***	0.51***	ns	0.21*
Controls	0.22*	ns	0.40***	0.32**	ns	0.22*
**U-Etiocholanolone**						
Athletes	0.23*	ns	0.49***	0.61***	0.26*	ns
Controls	ns	ns	0.29**	0.33**	ns	0.26*
**U- 5α Adiol**						
Athletes	0.35***	0.27**	0.40***	0.62***	ns	0.24*
Controls	0.32**	0.26*	0.31**	0.49***	0.30**	0.37***
**U- 5β Adiol**						
Athletes	0.21*	ns	0.35***	0.51***	0.21*	ns
Controls	ns	ns	ns	0.22*	ns	ns

*Values reported are Spearman rank-order correlation (*r*_*s*_).*

*E1, estrone; E2, estradiol; FAI, free androgen index; FSH, follicle-stimulating hormone; LH, luteinizing hormone; ns, non-significant; T, testosterone; U-5αAdiol, U-5α-Androstane-3α, 17β-diol; U-5βAdiol, U-5β-Androstane-3α, 17β-diol.*

^#^del/del (*n* = 8 controls, *n* = 9 athletes) excluded from analyses.

*<0.05; **<0.01; ***<0.001.

### Correlations Between Training Hours per Week, Serum and Urinary Steroid Metabolites

**FIGURE 1:**
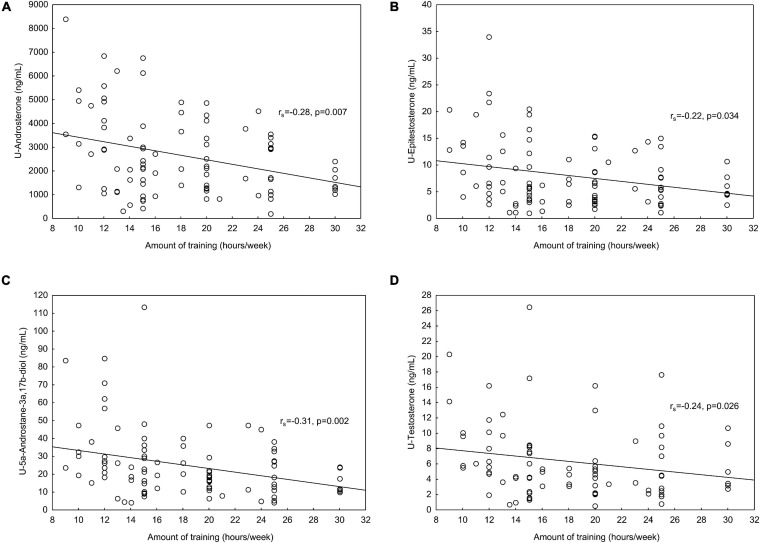
**(A)** In the athletes, significant negative correlations were found between amount of training (hours/week) and **(A)** U-Androsterone, **(B)** U-Epitestosterone, **(C)** U-5αAdiol (U-5α-Androstane-3α, 17β-diol) and **(D)** U-testosterone. In the U-testosterone correlations, UGT2B17 del/del individuals (*n* = 9) were excluded from the analyses.

In the athletes, significant negative correlations were found between training hours per week and U-Androsterone, U-Epitestosterone, U-5αAdiol, and U-testosterone, respectively (Figure 1). One of the ratios included in the ABP, A/Etio, also correlated negatively with training hours per week (*r*_*s*_ = −0.22, *p* = 0.036). No significant correlations were found between serum androgens and trainings hours per week.

## Discussion

For the first time, we demonstrate a difference in urinary steroid levels between female athletes and sedentary controls, i.e., the urinary levels of steroid metabolites both glucuronide and sulfate conjugated, were lower in the athlete population. Similar findings have previously been described when comparing male athletes and controls (Timon et al., 2008). Since it has been shown that exercise acutely increases the production and serum concentrations of androgens, lower levels of all the ABP metabolites may appear contradictory. Stress has been discussed as an influencing factor that may increase the excretion rate of urinary steroids IC, particularly in women (Mullen et al., 2020). There was no difference in cortisol levels between the sedentary controls and the athletes, however, when HC users were excluded, cortisol levels were higher in the athletes, and hence the influence of stress cannot explain the lower levels of steroid metabolites in the athletes.

The consistently lower urinary steroid levels in the athletes are not reflective of the serum steroids. In our participants, there were no differences in circulatory levels of any of the ABP related androgens studied between groups. In contrast, as previously published in the total cohort of Swedish female Olympic athletes, the serum androgen precursor dehydroepiandrosterone (DHEA) was higher in the athletes compared to controls (Eklund et al., 2017). A possible explanation may be that androgens in athletes are also eliminated by additional routes, i.e., in feces and/or sweat. However, quantification of steroids in feces have been poorly studied in humans. In eight healthy men, Colldén et al. (2019) showed that T and DHT are highly abundant in feces. Sweat might be a potential excretion route due to the lipophilic properties of steroids (Thieme, 2012). Already in 1983, it was reported that steroid metabolites included in the ABP (A and T) can be excreted as sulphate conjugates in human axillary sweat (Tóth and Faredin, 1985), whereas the glucuronide conjugated metabolites have not been evaluated in sweat. It is unlikely that different phase II metabolism explain the different urinary excretion rate of ABP markers between athletes and sedentary controls as the androgen-sulfates levels were also higher in the controls. It is therefore possible that sweat excretion might at least partly explain the negative correlations between amount of training (hours) and urinary levels observed herein.

After transdermal application of estr-4-ene diol, the metabolites nortestosterone, and estr-4-enedione were found in sweat collected after physical exercise (Thieme et al., 2003). The sweat production may depend on training intensity, sport activity, and temperature, etc. No difference between sport categories could be discerned (data not shown), possibly due to lack of power. As the ratios (particularly T/E) rather than the metabolites are monitored in the ABP, the non-urinary excretion routes may not have a direct impact on doping testing. However, it is possible that training mediated fluctuations in absolute androgen concentrations may be visible in an athlete’s passport, particularly in connection to situations where large differences in training load are expected, i.e., between in and off-seasons and after injuries. A future longitudinal study with controlled training schedule and analyses of additional sample matrixes (sweat) would be of interest to understand the connection between amount of training and urinary excretion rates of androgens. Moreover, sweat has been discussed as an alternative matrix in forensic toxicology, including detection of steroid abuse (Thieme, 2012).

The knowledge on how urine and serum androgen metabolites are connected may be of interest in anti-doping since quantification of endogenous serum steroids may be a complementary approach to the urinary steroid profile method in the future (Salamin et al., 2020). The monitoring of serum T may increase the likelihood to detect T intake in female athletes as serum T is superior compared to the urinary steroid profile (Handelsman and Bermon, 2019; Börjesson et al., 2020; Knutsson et al., 2020). Our correlations between the urinary ABP metabolites and serum hormones are in agreement with previously published data (Knutsson et al., 2020; Schulze et al., 2020).

Certain limitation of the present study should be addressed. Due to the cross-sectional study design, causality cannot be concluded. Even though blood and urine samples were collected by a standardized procedure (in a fasted and over-night rested state), we acknowledge that the sampling was performed randomly according to the menstrual cycle. In premenopausal women, there is a small mid-cycle increase in serum T (Handelsman et al., 2018). In addition, urinary androgens, especially urinary E, fluctuates during the menstrual cycle (Schulze et al., 2020). We found that urinary E demonstrated the strongest association with serum estrogens. Subsequently E is more sensitive to menstrual cycle fluctuations than other urinary metabolites (Schulze et al., 2020), resulting in larger individual ABP ranges in women than in men (Mullen et al., 2020). However, since doping tests are collected randomly, we believe that the results presented here provide valuable information.

It is well known that HC use affects serum T and SHBG levels (Sonalkar et al., 2000; Zimmerman et al., 2014). Furthermore, urinary androgens vary depending on HC use. In a previous study including female athletes, we showed that urinary E was suppressed in HC users (Schulze et al., 2014). These findings were confirmed in an intervention study examining the disposition of the androgen metabolites and ABP ratios in relation to HC use (Ekström et al., 2019). In the current study, the correlation analyses between serum and urinary androgens were evaluated in both HC and non-HC users. When HC-users were excluded, we did not observe any significant difference in the associations between serum- and urine androgens. Therefore, we suggest that regardless of HC use, the urinary metabolites reflect the androgenic load (serum concentrations of T, FAI, and LH) to the same degree in both athletes and controls. Furthermore, we found comparable differences in urinary steroid levels between athletes and controls in the subgroup not using HC.

It is well known that UGT2B17 exerts a large impact on the urinary concentrations of T (i.e., T-glucuronide). Therefore, we excluded the del/del subjects from the statistical analyses including urinary T. The UGT2B17 deletion polymorphism was found in same frequency in athletes and controls, i.e., approximately 10% being homozygous for the deletion allele. This allele frequency corroborates with other studies conducted in samples from athletes (Anielski et al., 2011; Choong et al., 2016). Another limitation is that training hours per week were based on self-reported data by the athletes.

In conclusion, we have shown that the urinary excretion rate of androgen metabolites monitored in ABP are higher in sedentary controls than in elite athletes, and that the amount of training is negatively associated with the urinary concentrations. Further studies are needed to understand the association between training and urinary excretion rate of androgens in athletes.

## Data Availability Statement

The raw data supporting the conclusion of this article will be made available by the authors, without undue reservation.

## Ethics Statement

The studies involving human participants were reviewed and approved by Regional Ethics Committee, Stockholm (EPN 2011/1426-32). The patients/participants provided their written informed consent to participate in this study.

## Author Contributions

AH, EE, and LE were involved in the concept and design of the study. AH and EE were responsible for the acquisition of data and in collaboration with LE and AA also the data analysis. AA performed the quantification of urinary androgens. EE, AA, LE, and AH were involved in the manuscript preparation, critical revision of the manuscript, and approval of the manuscript. All authors listed met the conditions required for full authorship.

## Conflict of Interest

AH is medical adviser to the Swedish Olympic Committee, the International Association of Athletic Federation and the International Olympic Committee. The remaining authors declare that the research was conducted in the absence of any commercial or financial relationships that could be construed as a potential conflict of interest.

## Publisher’s Note

All claims expressed in this article are solely those of the authors and do not necessarily represent those of their affiliated organizations, or those of the publisher, the editors and the reviewers. Any product that may be evaluated in this article, or claim that may be made by its manufacturer, is not guaranteed or endorsed by the publisher.
