# Supplementary material for: Urinary Steroid Profile in Elite Female Athletes in Relation to Serum Androgens and in Comparison With Untrained Controls
Source: Front Physiol. 2021 Aug 30;12:702305. doi: 10.3389/fphys.2021.702305 (PMC8435769; doi:10.3389/fphys.2021.702305)
Supplement: Supplementary file 1 [file Table_1.DOCX]

Supplementary Material

# Supplementary Table 1

| **Table 1.** Urinary androgen metabolites in female Olympic athletes and controls not using hormonal contraception. | | |
| --- | --- | --- |
| **U- androgen metabolites** | **Controls** | **Athletes** |
| n | 55 | 58 |
| U- Testosterone (ng/mL) | 7.00 (4.27-16.33) | 5.10 (2.75-9.05)* |
| U- Epitestosterone (ng/mL) | 14.93 (10.41-24.01) | 8.80 (4.78-13.45)*** |
| U- Androsterone (ng/mL) | 3397 (2079-6191) | 2418 (1313 -3666)** |
| U- Etiocholanolone (ng/mL) | 3963 (2592-5575) | 2864 (2034 -4843)* |
| U- 5αAdiol (ng/mL) | 36.1 (24.4-64.2) | 23.6 (15.9-35.9)*** |
| U- 5βAdiol (ng/mL) | 85.9 (51.6-186.9) | 93.2 (41.8-144.7) |
| T:E ratio | 0.6 (0.4-0.9) | 0.7 (0.3-1.2) |
| A:Etio ratio | 1.0 (0.8-1.2) | 0.8 (0.6-1.2) |
| A:T ratio | 458 (294-647) | 407 (287-696) |
| 5αAdiol:E | 2.5 (1.5-3.9) | 3.0 (1.8-4.3) |
| 5αAdiol:5βAdiol | 0.4 (0.2-0.7) | 0.3 (0.2-0.6)* |
| Values presented as median and interquartile range (25^th^-75^th^ percentile).  5αAdiol=U-5α-Androstane-3α,17β-diol, 5βAdiol=U-5β-Androstane-3α,17β-diol, A=androsterone, E=epitestosterone, Etio=etiocholanolone, T=testosterone.  *p < 0.05, **p < 0.01, ***p < 0.001. | | |
